# Excitability of the motor cortex in patients with migraine changes with the time elapsed from the last attack

**DOI:** 10.1186/s10194-016-0712-z

**Published:** 2017-01-06

**Authors:** Francesca Cortese, Gianluca Coppola, Davide Di Lenola, Mariano Serrao, Cherubino Di Lorenzo, Vincenzo Parisi, Francesco Pierelli

**Affiliations:** 1 Department of Medico-Surgical Sciences and Biotechnologies, ‘Sapienza’ University of Rome Polo Pontino, Corso della Repubblica 79, 04100 Latina, Italy; 2 G. B. Bietti Foundation IRCCS, Research Unit of Neurophysiology of Vision and Neuro-Ophthalmology, Rome, Italy; 3 Don Carlo Gnocchi, Onlus Foundation, Milan, Italy; 4 INM Neuromed IRCCS, Pozzilli, (IS) Italy

**Keywords:** Migraine, Transcranial magnetic stimulation, Motor threshold, Interictal, Ictal

## Abstract

**Background:**

Motor-evoked potentials (MEPs) produced by single-pulse transcranial magnetic stimulation (TMS) of the motor cortex can be an objective measure of cortical excitability. Previously, MEP thresholds were found to be normal, increased, or even reduced in patients with migraine. In the present study, we determined whether the level of cortical excitability changes with the time interval from the last migraine attack, thereby accounting for the inconsistencies in previous reports.

**Methods:**

Twenty-six patients with untreated migraine without aura (MO) underwent a MEP study between attacks. Their data were then compared to the MEP data collected from a group of 24 healthy volunteers (HVs). During the experiment, the TMS figure-of-eight coil was positioned over the left motor area. After identifying the resting motor threshold (RMT), we delivered 10 single TMS pulses (rate: 0.1 Hz, intensity: 120% of the RMT) and averaged the resulting MEP amplitudes.

**Results:**

The mean RMTs and MEP amplitudes were not significantly different between the MO and HV groups. In patients with MO, the RMTs were negatively correlated with the number of days elapsed since the last migraine attack (rho = -0.404, *p* = 0.04).

**Conclusion:**

Our results suggest that the threshold for evoking MEPs is influenced by the proximity of an attack; specifically, the threshold is lower when a long time interval has passed after an attack, and is higher (within the range of normative values) when measured close to an attack. These dynamic RMT variations resemble those we reported previously for visual and somatosensory evoked potentials and may represent time-dependent plastic changes in brain excitability in relation to the migraine cycle.

## Background

Although the pathophysiology of migraine remains unclear, neurophysiological studies performed over the last few decades have shown that patients affected by migraine exhibit interictal abnormalities in their cortical information processing system [1, 2]. These functional brain abnormalities are not constant; rather, they cyclically change until an attack occurs, whereupon the cortical responsiveness normalises [3]. The latter was demonstrated when information processing was assessed by cortical evoked potentials (EPs). In fact, the migraineur brain is frequently characterised by abnormal EP amplitude habituation in response to any kind of sensory stimulation [3]. We recently found that in migraineurs, the degree of EP abnormalities fluctuates over time, particularly in relation to the occurrence of migraine attacks (i.e. the degree of abnormalities is higher at long time intervals after an attack while it is minimal and within the normal range during an attack) [4–6].

Cortical excitability can also be examined non-invasively by applying transcranial magnetic stimulation (TMS) pulses over different areas of the cortex and then recording the evoked peripheral activity. TMS studies of the motor cortex rely on an objective measure, namely the motor-evoked potentials (MEPs) recorded from the peripheral muscles. In clinical practice and in scientific studies, corticospinal excitability is estimated objectively by examining the cortical motor threshold (or resting motor threshold, RMT), which is the minimal intensity of motor cortex stimulation required to elicit a MEP of minimal amplitude in the relaxed target muscle. The MEP size or amplitude can then be measured by setting the TMS intensity to 115–125% of the individual’s RMT [7]. Lower MEP thresholds and larger MEP amplitudes suggest higher cortical excitability. In patients with migraine, controversial findings have been reported regarding the degree of motor cortex excitability. Globally, thresholds for MEPs were found to be normal [8–13], increased [14–16], or reduced [17–19] in migraineurs. However, whether these inconsistent findings result from variation in the cortical excitability related to the time interval between the ictal and interictal state remains unknown.

Here, we sought to understand whether the actual MEP threshold and amplitude in patients with migraine varies on the basis of the time elapsed since the last attack and in comparison to healthy volunteers (HVs). Consistent with the abovementioned changes in EP according to the time elapsed from the last attack [4, 6], we hypothesised that motor cortex excitability would also become increasingly abnormal in patients with migraine as the time from the last migraine attack increased.

## Methods

### Participants

Thirty-one patients affected by migraine without aura (MO) who consecutively attended the Headache Clinic of the ‘Sapienza’ University of Rome Polo Pontino, Italy, were enrolled in this study. Only the data from patients who had an interval of at least 3 days between the recording and their last or next migraine attack (checked by email or telephone) were included. We also excluded those participants who were taking any type of medication on a regular basis, except contraceptive pills.

We evaluated the following clinical characteristics of the patients: duration of migraine disease (years), attack frequency (number/month), attack duration (hours), severity of headache attacks (0–10), and number of days elapsed since the last migraine attack (Table 1). This information was collected from participants’ 1-month headache diaries, which were obtained either during the screening visit or on the day of the recording session.

**Table 1:** Clinical and demographic characteristics of HVs and MO patients. Data are expressed as means ± SD

	HV (*n* = 24)	MO (*n* = 26)
Women (n)	16	18
Age (years)	30.4 ± 10.2	29.4 ± 6.8
Duration of migraine history (years)		13.9 ± 6.9
Attack frequency/month (n)		3.1 ± 2.7
Attack duration (hours)		22.3 ± 18.8
Visual analogue scale (n)		7.4 ± 1.5
Days from last migraine attack (n)		10.6 ± 8.4

Twenty-four HVs with a similar age and sex distribution as the patients with MO (mean age ± standard deviation: 30.4 ± 10.2 years, 16 women) and without a personal or familial history of migraine or any detectable medical condition were used for comparison. All participants were right-handed.

The physicians and neurophysiologists involved in the study were blinded to the electrophysiology and clinical history of the participants, respectively. This study was conducted in accordance with the Declaration of Helsinki and the study was approved by the Ethical Committee of the ‘Sapienza’ University of Rome Polo Pontino. All individuals provided written informed consent to participate in the study.

### Transcranial magnetic stimulation procedures

TMS was delivered through a high-frequency biphasic magnetic stimulator (MagstimRapid, The Magstim Company Ltd., Whitland, South West Wales, UK), which was connected to a figure-of-eight coil with a maximal output of 1.2 Tesla. Firstly, we determined the optimal orientation and position of the coil (i.e. ‘hot spot’) over the left motor area for stimulating the first dorsal interosseous muscle. After that, we identified the RMT by using single TMS pulses; complete relaxation of the first dorsal interosseous muscle was checked by verifying the absence of electromyographic signals, both visually (on a monitor) and by acoustic feedback. The RMT was defined as the minimal intensity required to elicit an electromyographic response of at least 50 μV with 50% probability in a fully relaxed muscle [7, 20–23].

During TMS, patients were seated in a comfortable armchair and asked to remain fully relaxed with their eyes closed to ensure similar attention levels. We delivered 10 single pulses of TMS (stimulus intensity: 120% of the RMT, rate: 0.1 Hz) and averaged the resulting MEPs.

### Statistical analysis

All analyses were conducted with the Statistical Package for the Social Sciences (SPSS) for Windows, version 21.0. The normality of the data for each group of participants was tested with the Shapiro–Wilk test. Since the MEP amplitude showed a non-Gaussian distribution, it was analysed with the non-parametric Mann-Whitney *U*-test. As the RMT was normally distributed, it was analysed using independent-samples *t*-tests. Spearman’s rho correlation test was used to search for correlations between the neurophysiological parameters and clinical variables mentioned above. Differences were considered statistically significant when the *p* value was <0.05.

## Results

**Fig. 1:**
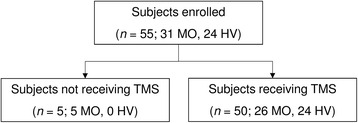
Flow chart showing the number of included/excluded participants in the various stages of the study

Among the 31 enrolled patients, five were excluded from the subsequent analyses because they had an attack during the hours after the recording session. Therefore, the final dataset consisted of 26 patients (Fig. 1).

**Fig. 2:**
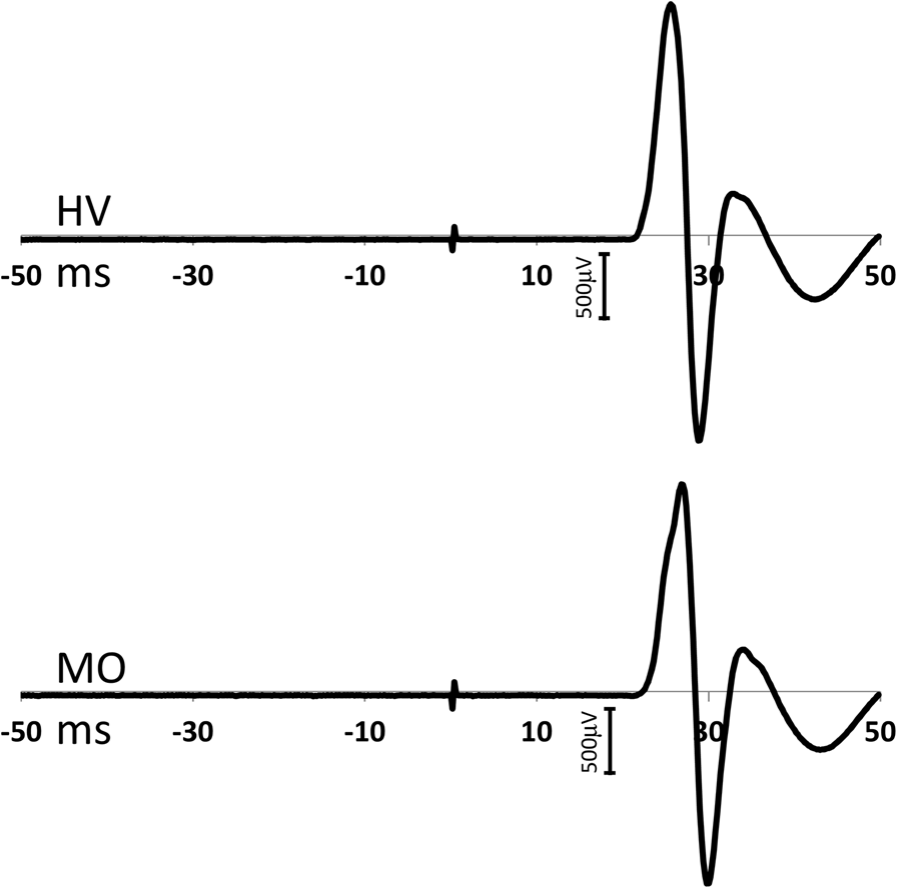
Trace illustrations of motor-evoked potentials (MEPs) from healthy volunteers (HVs) and patients with migraine without aura (MO)

The participant demographics and clinical characteristics of the MO group are listed in Table 1. Assessable MEP recordings were obtained from all participants. Examples of MEP recordings from participants in the HV and MO groups are shown in Fig. 2.

**Fig. 3:**
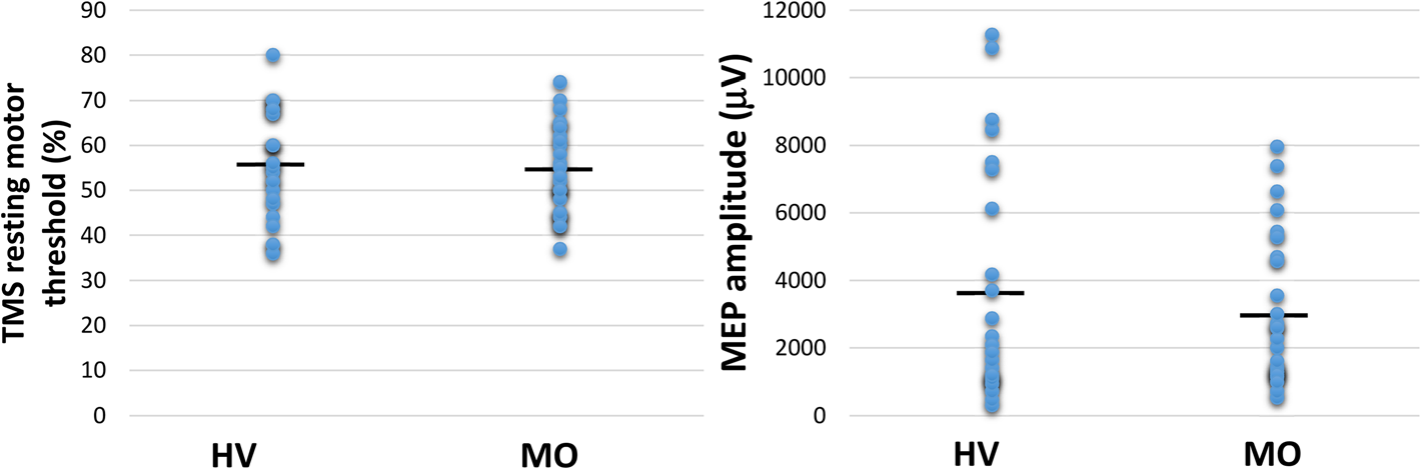
Grouped scatter-plot showing the resting motor thresholds (RMT [%]; left panel) and motor-evoked potential (MEP) amplitudes (right panel) in healthy volunteers (HVs) and patients with migraine without aura (MO)

No differences in interictal RMTs or MEP amplitudes were noted between the two participant groups (*t* = 0.536, *p* = 0.594 and *U* = 305.0, *p* = 0.892, respectively; Fig. 3).

**Fig. 4:**
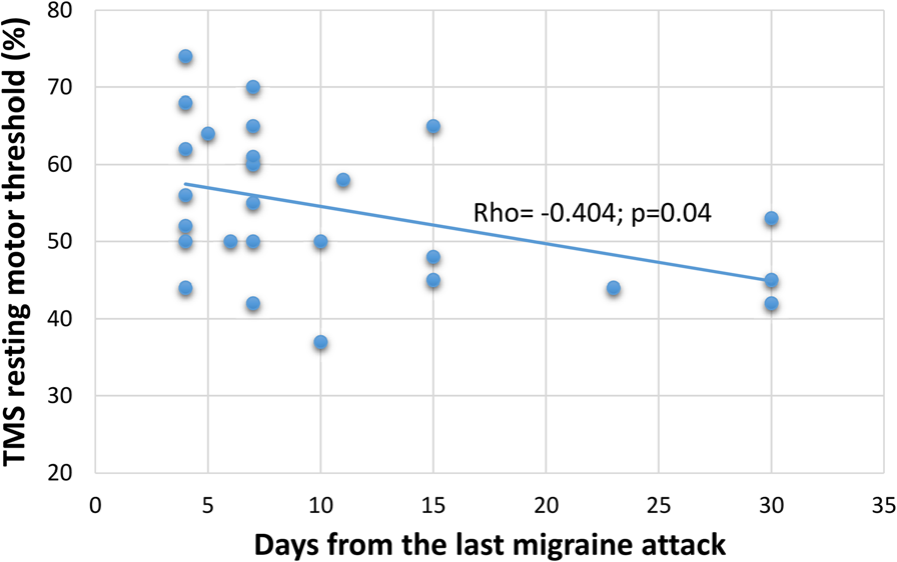
Correlation between the number of days since the last migraine attack and the resting motor threshold (RMT [%]) in patients with migraine who were between attacks

Spearman’s test revealed correlations between the neurophysiological parameters and clinical variables. In the MO group, the RMT was negatively correlated with the number of days since the last migraine attack (*rho* = -0.404, *p* = 0.04; Fig. 4). No other significant correlations were identified between the neurophysiological and clinical data in patients with MO.

## Discussion

Many clinical neurophysiology studies have shown that when patients with migraine are between attacks, their cortical responsiveness during the repetition of a series of stereotyped stimuli is enhanced when compared to controls. This functional brain abnormality has been detected in EPs for virtually all sensory modalities [3]. As mentioned earlier, previous single-pulse TMS studies examining motor cortex excitability in patients with migraine reported conflicting results. Overall, the results of the present study are concurrent with those of previous studies showing that the interictal RMTs and MEP amplitudes of patients with migraine do not differ from those of HVs [8–13].

To our knowledge, our study is the first to report a negative correlation between the RMT and time elapsed from the last migraine attack in patients with MO. This findings is consistent with previous evidence obtained with psychophysiological tests [24], neuroimaging techniques [25, 26], and cortical EPs [4–6] showing that during the variable pain-free period between two migraine attacks, the brain of an individual with migraine is exposed to subtle cyclic functional changes. Indeed, at the cortical level, we previously observed that patients with MO and a subgroup of patients with migraine with visual aura associated with paraesthesia and/or dysphasia exhibited a strong decrease in EP amplitude habituation during the stereotyped presentation of visual stimuli with the passing of time from the last attack [4, 5]. The results of the present study revealed that the same correlation is valid for the resting excitability of the motor cortex in response to single-pulse TMS. This finding indicates that motor cortex excitability fluctuates during interictal phases; specifically, as the time elapsed from the last attack increases so does the motor cortex disexcitability. These results are in favour of a migraine cycle-dependent subtle imbalance between excitation and inhibition in the motor cortex. Below, we discuss the possible neurophysiological underpinnings of these TMS results and their relevance to migraine pathophysiology.

TMS is a non-invasive technique that permits researchers to objectively evaluate the RMT and estimate motor cortex excitability [7]. At the RMT, TMS indirectly activates the pyramidal tracts by eliciting so-called indirect waves (I-waves), which result from the complex interactions among different types of cortical cells that discharge at a high frequency [27–29]. Modelling studies have shown that when the coil is placed tangentially on the scalp—as was the case here—the majority of the induced current flows parallel to the surface of the brain rather than perpendicular to the grey matter [30]. Consequently, TMS-induced horizontal current flow preferentially activates the horizontally oriented axons of cortical interneurons or cortico-cortical fibres that activate pyramidal neurons trans-synaptically (I-waves) instead of activating pyramidal neurons directly (D-waves). Therefore, the excitation threshold depends on the orientation and membrane properties of the axons activated by the TMS-induced electrical field, including axons of the tangentially oriented cortico-cortical loop fibres that modulate the excitability of the corticospinal output neurons.

Among the cortico-cortical fibre systems, it is important to consider the influence that collateral gamma-aminobutyric acid (GABA)-ergic axons, which project from the somatosensory cortex, have on motor cortex excitability, as shown in animal studies [31, 32] and in human studies using paired associative stimulation [33]. Moreover, it is well known that cortico-cortical loops, particularly in the general and somatosensory cortices, are strongly modulated by thalamocortical afferent fibres [32]. Interestingly, somatosensory lateral inhibition and thalamocortical drives are both involved in the pathophysiology of interictal migraine. Early somatosensory high-frequency oscillation bursts (detected by the appropriate filtration of common somatosensory evoked potentials), which reflect thalamocortical spike activity, are reduced in episodic migraine interictally; however, they normalise during an attack [34]. The microstructural correlates of these thalamic functional fluctuations were recently investigated in a diffusion tensor magnetic resonance study [26], which found that the interictal fractional anisotropy was significantly increased while the mean diffusivity was slightly decreased within the thalamus bilaterally. Interestingly, the right thalamic fractional anisotropy was positively correlated with the number of days since the last migraine attack, which is consistent with the results of the present study. Furthermore, a recent neurophysiological study [6] showed that patients with migraine have deficient lateral inhibition within the somatosensory cortex during the interictal phase; however, they show normal lateral inhibition during the attack. Nonetheless, the degree of somatosensory lateral inhibition is directly related to the somatosensory thalamocortical activity (evaluated as the amplitude of presynaptic high-frequency oscillations) and inversely related to the number of days elapsed since the last attack [6].

Owing to this interictal, morphofunctional thalamocortico-cortical evidence in patients with migraine, we postulate that the reduced thalamic control of the sensorimotor cortical activity and decreased degree of somatosensory lateral inhibition, which are both inversely correlated with the number of days since the last attack, could account for the observed subtle fluctuations in the RMT during the variable pain-free period between migraine attacks. However, whether these abnormalities in sensorimotor cortical activity are consequences of the ‘thalamocortical dysrhythmia’ [35, 36] (a model theory on cyclical functional abnormalities in migraine) remains unknown. Regardless, in a previous study on a group of mixed patients and HVs, we found that inhibitory TMS-induced plastic changes were inversely related to the level of thalamocortical activation [37], supporting the hypothesis that anomalous thalamic control could underlie the abnormal TMS findings in patients with migraine who are between attacks.

Finally, we acknowledge as a possible limitation of the present study that some researchers observed that the RMT was not stable over days, which may have complicated the interpretation of values measured at one point in time [38]. However, this is not completely detrimental because it may further support our findings that cortex excitability is not stable between attacks but rather undergoes daily fluctuations during the so-called migraine cycle.

## Conclusions

Here, in patients with MO who were between attacks, we detected a negative correlation between the RMT and the number of days since the last attack. Our results help explain the conflicting findings reported previously on the degree of motor cortex excitability in patients with migraine by showing that the RMT is strongly dependent on the phase of the migraine cycle. We propose that hypofunctioning of the thalamocortical loops and somatosensory lateral inhibition, beyond accounting for the dynamic variations in the sensory cortex habituation deficits, may contribute to the observed subtle fluctuations in motor cortex excitability in patients with migraine. We believe this occurs by influencing the cortico-cortical GABAergic inhibitory connections between the somatosensory and motor cortical areas. Further studies are needed to determine whether interactions among sensory and motor cortical activity under the control of thalamic nuclei are involved in the clinical and morphofunctional features of patients with migraine, including those experiencing aura or headache chronification.

## Abbreviations

D-wave: Direct wave
EP: Evoked potential
GABA: Gamma-aminobutyric acid
HV: Healthy volunteer
I-wave: Indirect wave
MEP: Motor-evoked potential
MO: Migraine without aura
RMT: Resting motor threshold
TMS: Transcranial magnetic stimulation
